# Parent’s Cardiorespiratory Fitness, Body Mass, and Chronic Disease Status Is Associated with Metabolic Syndrome in Young Adults: A Preliminary Study

**DOI:** 10.3390/ijerph16101768

**Published:** 2019-05-19

**Authors:** Paul B. Nolan, Graeme Carrick-Ranson, James W. Stinear, Stacey A. Reading, Lance C. Dalleck

**Affiliations:** 1 Exercise Science, College of Nursing and Health Science, Flinders University, Adelaide 5001, Australia; 2 Exercise and Sport Science, School of Health Sciences, University of South Australia, Adelaide 5001, Australia; Graeme.carrick-ranson@unisa.edu.au; 3 Department of Exercise Sciences, Faculty of Science, The University of Auckland, Auckland 1023, New Zealand; j.stinear@auckland.ac.nz (J.W.S.); s.reading@auckland.ac.nz (S.A.R.); 4 Recreational, Exercise, and Sport Science Department, Western Colorado University, Gunnison, CO 81231, USA; ldalleck@western.edu

**Keywords:** metabolic syndrome, young adult, primordial prevention, primary prevention, cardiovascular disease screening

## Abstract

We sought to determine if there was an intergenerational association between parental weight, cardiorespiratory fitness (CRF), and disease status, with the prevalence of metabolic syndrome (MetSyn) in their young adult offspring. Young adults (*n* = 270, 21 ± 1 years, 53.3% female) were assessed for MetSyn and self-reported parent’s CRF, body mass status, and disease status. MetSyn was present in 11.9% of participants, 27.4% had one or two components, and 58.5% had no components. A significantly higher percentage (93.9%) of young adults with MetSyn identified at least one parent as being overweight or obese, 84.8% reported low parental CRF and 87.9% reported a parent with disease (all *p* < 0.017). MetSyn in offspring is more likely when parents are perceived to have low CRF, increased body mass, and a diagnosis of disease. Evaluating the offspring of people with low CRF, elevated body mass, or who have a history of cardiovascular disease (CVD) or diabetes should be considered to promote early identification and treatment of young adults to reduce future premature CVD in these at-risk individuals.

## 1. Introduction

Metabolic Syndrome (MetSyn) encompasses a broad range of metabolic abnormalities [1], which is reported to be present in 20–30% of adults worldwide [2]. MetSyn prevalence varies with age and is present in 5–7% of apparently healthy young adults worldwide [3]. There is a two-fold increase in the risk of developing cardiovascular disease (CVD) and a five-fold increase in the risk of developing type 2 diabetes mellitus in people with MetSyn [4]. Early identification of the presence of MetSyn allows for a greater opportunity of reversal and prevention of subsequent clinical disease, highlighting the clinical importance of early identification of MetSyn to reduce lifetime disease risk.

Lifestyle interventions focusing on increasing physical activity levels, improving diet, and weight loss are effective in reducing MetSyn in young adults [5,6]. However, the identification of young adults who have a higher risk of MetSyn and, therefore, the development of clinical disease is difficult. For example, despite cholesterol screening being recommended for adults from the age of 20 years [6], less than 50% of 20–35-year-old males and 20–45-year-old females participate in cholesterol screening. Furthermore, rates of cholesterol screening appear to be no different in young adults with differing levels of CVD risk factors [7]. Thus, there appears to be a clear need for new approaches to identify young adults with a high lifetime CVD risk in order to prevent future CVD.

There is clear evidence of intergenerational links for CVD and CVD risk factors between parents and offspring [8,9,10]. Moreover, one of the traditional CVD risk factors is having a father with diagnosed CVD under the age of 55 years or having a mother with diagnosed CVD under the age of 65 years [11]. Despite this knowledge, studies that have specifically looked at the association between MetSyn in the offspring of people with a diagnosis of CVD or diabetes are rare. Furthermore, there is sparse information on the association between parent’s lifestyle and their offspring’s MetSyn status. Therefore, the aim of this preliminary study was to determine the association between perceived parent cardiorespiratory fitness (CRF), body mass, and disease status, and the presence of MetSyn in young adults. We hypothesised that parents of young adults with MetS are more likely to have low CRF, be overweight or obese, and have either CVD or diabetes.

## 2. Materials and Methods

Young adult (18–25 years) participants (*n* = 270, 53.3% female) were recruited from Colorado, the United States of America (*n* = 139), and Auckland, New Zealand (*n* = 131). Participant recruitment was performed using a combination of poster advertisement and in-lecture presentation to large undergraduate classes at the University of Auckland and Western State Colorado University. Participants that had known underlying metabolic conditions, such as polycystic ovary syndrome, type 2 diabetes mellitus, or were pregnant or lactating at the time of assessment, were excluded from this study. Participants attended the testing laboratory after an overnight fast, avoidance of vigorous physical activity in the preceding 24 h, and abstinence from caffeine or tobacco in the preceding four hours. After obtaining informed consent, participants were asked to answer closed yes or no questions regarding their perception of their parent’s CRF, body mass, CVD, and diabetes status (see Table 1). Participants were instructed to answer yes, if either of their parents were obese, if either of their parents were overweight, and instructed to answer yes if either parent, in their opinion, had good CRF.

Subsequent to the informed consent process and questionnaire (approximately 10 min of seated rest), resting blood pressure (BP) and heart rate (HR) were recorded with the participant’s feet on the floor and arm supported at heart level with an automated sphygmomanometer (Life Brand, Toronto, ON, Canada; Omron HEM-705CP, Bannockburn, IL, USA).

### 2.1. Total Body Mass, Height and Waist Circumstance

Participant’s height and total body mass were recorded to the nearest 0.1 kg and 0.1 cm in duplicate using calibrated digital scales (SECA770, Germany) and a standard stadiometer (SECA217, Germany).

Waist circumference (WC) was measured in the standing position at the narrowest point between the iliac crest and the 11th and 12th ribs using either a metal tape (Lufkin W606PM, Apex tool group LLC, Sparks, MD, USA) or cloth tape with spring loaded handle (Creative Health Products, Ann Arbor, MI, USA).

BP, HR, total body mass, height, and WC were measured in duplicate with the mean of the two measures recorded as the final datum point. When two measures were outside the acceptable range (BP ± 5 mmHg, HR ± 5 bpm, WC ± 1.0 cm, mass ± 0.1 kg, height ± 1 cm), a third measure was performed and the median of the three measurements was recorded as the final datum point.

### 2.2. Blood Measures

Blood lipid and glucose measures were analysed via a Cholestech LDX System (Alere Inc., Waltham, MA, USA). A finger prick was made on the participant’s ring finger of their non-dominant hand using aseptic techniques. Blood was sampled using a small capillary tube that was inserted into a preloaded cassette for immediate analysis. Triglyceride (TG) level, high-density lipoprotein cholesterol (HDL-C), low-density lipoprotein cholesterol (LDL-C), total cholesterol, and fasting blood glucose (FBG) were directly measured or estimated using the Friedewald formula. When blood lipids were outside the detectable upper or lower limit of the Cholestech LDX System (*n* = 33), the last detectable value for that variable was substituted. This procedure does not affect the ability to detect MetSyn components.

### 2.3. Participant Classification

MetSyn component data (BP, HDL-C, TG, FBG, and WC) collected from participants were evaluated against harmonised MetSyn criteria [4]. Participants were stratified as healthy if no components were present, at-risk if one or two components were present, and present if three or more components were present.

### 2.4. Statistical Analysis

A one-way ANOVA, adjusted for sex and site of assessment, was performed to determine differences in participant characteristics across the three groups (present, at-risk, and healthy). To determine specific group differences, post-hoc pairwise analysis utilizing a Bonferroni correction for multiple comparisons were performed if statistically significant results were present.

To determine the association between parent’s CRF, parent’s body mass, and parent’s chronic disease status, a series of chi-square analyses was performed. When significant results were present, post-hoc z-column proportion analysis utilizing a Bonferroni correction for multiple comparisons was performed to determine specific group differences.

Statistical significance was set at *p* < 0.05 for the one-way ANOVA and chi-square analysis. Where multiple comparison occurred in post-hoc testing, a Bonferroni correction was applied. Therefore, statistical significance was set at *p* < 0.017 for post-hoc testing of all analyses involving the healthy, at-risk, and present groups.

All analyses were performed using IBM SPSS statistics version 25 (IBM Corporation, New York, NY, USA).

All study protocols were approved by the relevant local ethics and institutional review boards (Auckland 012544, Colorado HRC2013-0261R3).

## 3. Results

One hundred and fifty eight participants (58.5%) had no MetSyn components (healthy), 74 participants (27.4%) had one or two components (at-risk), and 32 participants (11.9%) had three or more components (present).

There was no between age group differences, and no differences in resting HR or estimated CRF between the at-risk and healthy groups. There was no significant difference in HDL-C in the present and at-risk groups. All other comparisons were statistically significant between groups (see Table 2).

### Association between Parent’s Status and Metabolic Syndrome Status

Table 3 displays the percentage of participants within each MetSyn group that had a parent with each characteristic. Many participants (*n* = 158) indicated at least one parent was overweight or obese. There was a significantly greater prevalence of parents with at least one parent who were overweight or obese in the present group (93.9%, *p* ≤ 0.0017). Furthermore, in the present group a similar result was seen in regards to parental CRF (15.2%, *p* ≤ 0.0017), and disease status (87.9%, *p* ≤ 0.0017).

The majority of young adults with MetSyn had a parent who had disease, was overweight, and was “unfit” (84.8% of present group). Conversely, the highest percentage of healthy participants reported parents did not have disease, were not overweight or obese, and one or more had good fitness (31.7%) (see Table 4).

## 4. Discussion

The main finding of this preliminary study is that there is an association between parental CRF, body mass status, chronic disease status, and young adult offspring’s MetSyn status. The current study strongly suggests that young adults with at least one parent who has low CRF, is overweight or obese, or has CVD or diabetes, may already have a detectable increase in their lifetime risk for CVD and diabetes. Fortunately, early intervention in the young adult offspring of people with low CRF, increased body mass, and CVD or diabetes may help curb development of MetSyn and the associated risk for future development of CVD and diabetes. Therefore, consideration of targeted cardiometabolic health screening in young adults who have parents with the characteristics described, may be warranted to break the cycle of intergenerational cardiometabolic disease.

Several large cohort epidemiological studies have shown a familial aggregation of CVD risk factors [8,9,12] with parental-offspring correlations being stronger than spousal correlations suggestive of a strong genetic component [12]. Moreover, there are reports of MetSyn aggregation between generations [13] with both genetic and environmental factors being influential [14]. The current findings support these previous reports and suggest that MetSyn is higher in young adults who self-report having a parent with CVD or diabetes than their peers who do not. Importantly, we provide preliminary evidence of a link between parental CVD and diabetes and an early pre-clinical syndrome (i.e., MetSyn). Therefore, to reduce future CVD rates, one public health strategy could be to assess offspring of people with new diagnoses of CVD or diabetes for CVD risk with the intention of providing primary or primordial prevention services.

In addition to established parental disease, the current findings also suggest that young adults who report a parent with low CRF or are overweight or obese have increased prevalence of MetSyn. The role of lifestyle in the development of CVD [15] is well established, so this finding is not unexpected. However, the association with parental lifestyle and their offspring’s MetSyn status is not clearly elucidated. Speculatively, in the current study many of the participants still resided with their parents and, therefore, the influence of parent’s lifestyle may have influenced the participant’s current lifestyle, therefore MetSyn status.

There are significant reductions in MetSyn with increases in CRF with exercise training [16,17], the association of parental fitness with MetSyn in their offspring is less well known. In the current study, there was a much lower prevalence of MetSyn in participants who reported having at least one parent with good CRF when considering participants who reported at least one parent who had increased body mass and had CVD or diabetes (3.0% good CRF parent vs. 84.8% poor CRF parent). While, we know unfortunately that due to the self-report methodology in the current study, we cannot rule out the possibility that participants were unable to discern the difference between CRF and fatness or may have perceived a parent with disease as automatically having poor CRF. However, given there is such a large difference in the percentage of young adults based on parent’s CRF status, future studies may wish to address this intriguing result.

### Limitations

The current study used a self-report questionnaire and parent’s CRF and body mass were not directly measured, and parents were not directly asked about their diagnosis of CVD or diabetes. Therefore, there may be some recall error on behalf of the participant and some parents may have been incorrectly classified. Furthermore, we did not collect information on the parent’s ages. However, despite these limitations in data collection about parents, we were still able to detect significant differences in the prevalence of MetSyn in participants. Furthermore, our results largely agree with previous research on intergenerational CVD risk factors. Therefore, we are confident that the results presented in this preliminary study would be replicated with a more robust, direct measurement of CRF, body mass, and disease status in young adult’s parents.

We did not collect information on which parent had poor CRF, increased body mass, CVD or diabetes. Furthermore, we categorised obese and overweight parents together and there may be different relationships in offspring health depending on the degree of parental mass. Collectively, further classification and direct measurement of parental characteristics may have helped strengthen associations. For example, there is evidence to suggest a stronger maternal effect on MetSyn components than paternal in adolescents [18], although this is not a consistent finding [9]. Further investigations should focus on these differences to establish the effect of maternal or paternal health on offspring cardiometabolic health.

## 5. Conclusions

We have provided preliminary evidence that parents perceived to have low CRF, increased body mass, and a diagnosis of CVD or diabetes are associated with offspring more likely to have MetSyn. While, larger studies with direct measurements of both parental and offspring mass, CRF, and disease status are required to confirm these preliminary findings, consideration should be given to evaluating the offspring of people with low CRF, increased body mass, or newly diagnosed CVD or diabetes patients, in order to reduce future premature CVD in these at-risk individuals. Providing early lifestyle intervention to reverse or modify known risk factors for chronic disease in young adults whom have a parent with CVD or diabetes may help to break the intergenerational cycle of CVD and diabetes.

## Figures and Tables

**Table 1:** Questions asked about parental cardiorespiratory fitness, body mass status, and diabetes and cardiovascular disease status.

Question
Do you consider your parents to be obese?
Do you consider your parents to be overweight?
Do you consider your parents to have good cardiorespiratory fitness?
Do either of your parents have diabetes?
Do either of your parents have heart disease (heart attack, stent, angina, bypass etc.)?

**Table 2:** Participant characteristics categorised by metabolic syndrome group.

Parameter	Healthy (*n* = 158)	At-Risk (*n* = 74)	Present (*n* = 32)
Age (years)	21 ± 1	21 ± 1	21 ± 1
Resting HR (bpm)	63 ± 9	66 ± 10	75 ± 6 ^b,c^
Resting SBP (mmHg)	114 ± 8	124 ± 11 ^a^	135 ± 6 ^b,c^
Resting DBP (mmHg)	70 ± 7	77 ± 9 ^a^	84 ± 5 ^b,c^
BMI (kg·m^−2^)	22.0 ± 2.9	24.4 ± 3.7 ^a^	30.3 ± 3.8 ^b,c^
WC (cm)	76.2 ± 8.3	80.0 ± 8.9 ^a^	97.8 ± 6.3 ^b,c^
TG (mmol·L^−1^)	0.90 ± 0.35	1.10 ± 0.53 ^a^	2.33 ± 0.68 ^b,c^
HDL-C (mmol·L^−1^)	1.60 ± 0.33	1.13 ± 0.35 ^a^	1.02 ± 0.13 ^b^
FBG (mmol·L^−1^)	4.70 ± 0.35	4.93 ± 0.44 ^a^	5.69 ± 0.30 ^b,c^

All data displayed as mean ± S.D.; ^a^ statistically significant difference (*p* ≤ 0.0017) between healthy and at-risk; ^b^ statistically significant difference (*p* ≤ 0.0017) between healthy and present groups; ^c^ statistically significant difference (*p* ≤ 0.0017) between at-risk and present groups. Healthy—no metabolic syndrome components; at-risk—one or two metabolic syndrome components; present—three or more metabolic syndrome components; HR—heart rate; SBP—systolic blood pressure; DBP—diastolic blood pressure; BMI—body mass index; WC—waist circumference; TG—triglyceride; HDL-C—high-density lipoprotein cholesterol; FBG—fasting blood glucose.

**Table 3:** Prevalence of participants in each metabolic syndrome group with a parent with or without poor cardiorespiratory fitness, increased body mass, or cardiometabolic disease.

Parameter	Healthy (*n*)	At-Risk (*n*)	Present (*n*)
**Parent’s mass status**			
≥1 overweight or obese	50.3% (81)	60.5% (46)	93.9% (31) ^a,b^
No overweight or obese	49.7% (80)	39.5% (30)	6.1% (2) ^a,b^
**Parent’s cardiorespiratory fitness status**			
≥1 good CRF	70.8% (114)	55.3% (42)	15.2% (5) ^a,b^
Both parents poor CRF	29.2% (47)	44.7% (34)	84.8% (28) ^a,b^
**Parent’s disease status**			
≥1 CVD or diabetes	27.3% (44)	30.3% (23)	87.9% (29) ^a,b^
No CVD or diabetes	72.7% (117)	69.7% (53)	12.1% (4) ^a,b^

^a^ statistically significant difference (*p* ≤ 0.0017) between healthy and present groups. ^b^ statistically significant difference (*p* ≤ 0.0017) between at-risk and present groups. Healthy—no metabolic syndrome components; at-risk—one or two metabolic syndrome components; present—three or more metabolic syndrome components; CVD—cardiovascular disease; ≥1—one or both.

**Table 4:** Association between all three parental characteristics and metabolic syndrome status.

Parental Status			Offspring Status		
Disease	Mass	CRF	Healthy (%)	At-Risk (%)	Present (%)
≥1 CVD or Diabetes	No overweight or obese	≥1 fit	8.7	2.6	0.0
		No fit	0.6	0	0.0
	≥1 overweight or obese	≥1 fit	8.1	5.3	3.0
		No fit	9.9	22.4	84.8
No CVD or diabetes	No overweight or obese	≥1 fit	31.7	26.3	6.1
		No fit	8.7	10.5	0.0
	≥1 overweight or obese	≥1 fit	22.4	21.1	6.1
		No fit	9.9	11.8	0.0

Healthy—no metabolic syndrome components; at-risk—one or two metabolic syndrome components; present—three or more metabolic syndrome components; CVD—cardiovascular disease; CRF—cardiorespiratory fitness; ≥1—one or both.
